# Predictability performance of urinary C–C motif chemokine ligand 14 and renal resistive index for persistent sepsis-associated acute kidney injury in ICU patients

**DOI:** 10.1007/s11255-023-03511-6

**Published:** 2023-02-17

**Authors:** Wei Jiang, Ting Liao, Jiangquan Yu, Jun Shao, Ruiqiang Zheng

**Affiliations:** 1 Clinical Medical College, YangZhou University, Yangzhou, 225001 China; 2 Intensive Care Unit, Northern Jiangsu People’s Hospital, Yangzhou, 225001 China

**Keywords:** Acute kidney injury, Sepsis, Persistent sepsis-associated acute kidney injury, Intensive care unit, Renal recovery, Renal resistive index, C–C motif chemokine ligand 14

## Abstract

**Objectives:**

The performance of renal resistance index (RRI) in predicting persistent sepsis-associated acute kidney injury (S-AKI) remains debatable, and the value of urinary C–C motif chemokine ligand 14 (CCL14) in predicting persistent S-AKI has not been validated yet. Therefore, we aimed to determine the applicability of a urinary biomarker CCL14 for the early detection of persistent S-AKI. Furthermore, the use of RRI obtained from renal Doppler ultrasonography was applied to differentiate transient from persistent S-AKI. Finally, we aimed to evaluate the use of these techniques in predicting different subtypes of S-AKI.

**Methods:**

This prospective observational study was conducted at the internal medicine intensive care unit (ICU) of a university hospital. The RRI was determined within 12 h of ICU admission and the urinary CCL14 was evaluated at T0, T6, T12, and T24. The reversibility of renal dysfunction was assessed within 48 h. The receiver operating characteristic curves were then plotted to assess the diagnostic efficacy of the RRI and urinary CCL14 in predicting persistent S-AKI.

**Results:**

Out of 48 patients, 23 developed persistent S-AKI upon admission. The RRI was higher in the persistent S-AKI group (*P* = 0.02) and the RRI ≥ 0.679 could predict persistent S-AKI with an area under the receiver operating characteristic curve of 0.79 (95% CI 0.65–0.93), a sensitivity of 91.30% (95% CI 70–98%), and a specificity of 65.20% (95% CI 43–83%). Urinary CCL14 was not significantly different between the two groups at the tested period, showing poor diagnostic performance at T0, T6, T12, and T24, with areas under the receiver operating characteristic curves of 0.56 (95% CI 0.38–0.73), 0.62 (95% CI 0.46–0.79), 0.52 (95% CI 0.35–0.68), and 0.60 (95% CI 0.43–0.77), respectively.

**Conclusions:**

The RRI obtained from renal Doppler ultrasound is extremely effective in predicting persistent S-AKI in critically ill patients, and urinary CCL14 could not distinguish between transient and persistent S-AKIs.

**Supplementary Information:**

The online version contains supplementary material available at 10.1007/s11255-023-03511-6.

## Introduction

Acute kidney injury (AKI) is a disease with a high global prevalence. Nearly 40% of its cases are commonly associated with sepsis [1, 2]. A recent study revealed that 68% of patients with sepsis had AKI on admission to the intensive care unit (ICU). Out of the 68% of patients, 40% had moderate-to-severe AKI and 27% had a severe condition for which they received continual renal replacement therapy (CRRT) [3]. Despite extensive research on the early detection of AKI, its duration and time frame studies remain unexplored [3]. The time duration of AKI is significantly important due to certain reasons. For instance, the duration of AKI is related to the patient's prognosis and the risk of end-stage renal failure. Recent evidence suggests that patients with persistent AKI have a significantly lower one-year survival rate [4] and they are at an increased risk of developing chronic kidney disease (CKD) when compared to transient patients [4, 5]. Timely monitoring can facilitate early detection of individuals at risk for persistent AKI, who can then receive active intervention for management, which can, in turn, potentially influence the transition of AKI to CKD [5, 6]. In addition, the AKI duration is associated with the line of treatment. It has been suggested that predicting the short-term reversibility of AKI may help to assess the likelihood of requiring CRRT [7, 8] and ultimately help determine the best time to initiate CRRT [5, 9–11]. Thus, the mode and method of intervention (CRRT) are based on the progression of the condition.

Apart from the time parameter, the detection of persistent AKI is equally important. Early detection can lead to precise treatment, thereby minimizing adverse conditions. Therefore, there is a need to develop a new diagnostic tool that can detect persistent AKI. Considering the importance of identifying persistent AKI, new diagnostic tools, including renal Doppler ultrasound and urinary biomarkers and have been evaluated for the identification of persistent AKI.

Recent studies depicted the use of renal resistance index (RRI) in predicting persistent AKI. RRI is a non-invasive and rapid tool used to differentiate transient and persistent AKI [12–15]. Several studies have demonstrated its validity in predicting persistent AKI [14–16]. This method seems promising, but the discrepancies between multiple data sets (such as in the number of patients and heterogeneity between studies) are concerning. These preliminary studies were performed in expert centers on small sample size. A meta-analysis highlighted the good performance of RRI in predicting persistent AKI but also revealed the great heterogeneity between the studies [17]. In fact, a recent multi-center study reported conflicting results with the previous ones, suggesting that RRI performs poorly in predicting persistent AKI. Moreover, RRI measurement has been reported to be dependent on multiple factors like vascular compliance, age, and intra-abdominal pressure [18–20], which affects its accuracy. Detection using different urinary biomarkers is also useful in predicting persistent AKI. A C–C motif chemokine ligand 14 (CCL14), a member of the chemokine small molecule family, was recently reported to predict persistent AKI. Hoste et al. found that urinary CCL14 was able to identify severe persistent AKI early [21], followed by external validation of CCL14 predicting persistent AKI in a study conducted by Sean M. Bagshaw et al.[22], which found that urinary CCL14 predicted persistent KIDGO stage 3 AKI with an Area Under Curve (AUC) was 0.81 [95% confidence interval (95% CI), 0.72–0.89], and the higher the urinary CCL14 value, the higher the risk of persistent KIDGO stage 3 AKI. It must, therefore, be acknowledged that both the discovery study by Hoste et al. and the external validation study by Bagshaw et al. have heterogeneity in their study populations only for patients with KIDGO stages II–III, and it is not known whether the same diagnostic efficacy of urinary CCL14 exists for different AKI subtypes (e.g., sepsis) as well as for patients with different AKI stages.

With this view, the current work focused on evaluating the use of RRI and CCL14 in effectively predicting persistent S-AKI.

## Patients and methods

### Patients

This prospective observational study involved screening sepsis patients admitted to the ICU of Northern Jiangsu People’s Hospital in Jiangsu Province, China, from November 1, 2020 to March 1, 2022. The study protocol was approved by the Ethics Committee of Northern Jiangsu People’s Hospital (2021ky007) and the registration was completed with the Chinese Clinical Trials Registry (ChiCTR2100050540).

Adult patients (age ≥ 18 years) who were diagnosed with sepsis on admission to the hospital and who developed AKI within 48 h of admission were included in the study based on the Sepsis-3 criteria and KIDGO criteria. The subject exclusion criteria were pregnancy, anuria, renal transplantation, chronic kidney disease stages IV–V, cardiac arrhythmias, stage C cirrhosis, obstructive nephropathy, AKI of glomerular etiology, and patients with a hospital stay of < 48 h.

### Definitions

The diagnostic criteria for AKI are an increase in the serum creatinine level ≥ 0.3 mg/dL (≥ 26.5 umol/L) within 48 h or an increase in the creatinine level ≥ 1.5-fold from the baseline values within 7 days of known disease onset or a sustained 6-h urine output < 0.5 mL/(kg-h) [23]. Within 48 h of inclusion, a decrease in the AKI severity of at least one stage according to the KDIGO criteria without diuretic as well as CRRT interventions was considered transient S-AKI [5]. Persistent S-AKI was defined as persistent oliguria and/or stable or higher AKI KDIGO stages. Sepsis and septic shock were diagnosed following the sepsis 3.0 criteria [24].

### Data collection and study protocol

Patient data extracted from the medical records included their demographic data, medical history, general clinical information (i.e., vital signs, routine blood, blood biochemical parameters, and blood gas analysis parameters), urine volume, and creatinine status at the time of inclusion, Sequential Organ Failure Assessment (SOFA) score, Simplified Acute Physiology Score II (SPAS II) score, vasoactive drug application, mechanical ventilation and CRRT, ICU length-of-stay, and ICU death.

For patients suspected of sepsis or diagnosed with sepsis at the time of admission to the ICU, 10 mL of urine specimens was retained for 6, 12, and 24 h in the ICU, followed by centrifugation at 3000 rpm for 5 min, and the supernatant was collected after 30 min of resting and stored at ≤ − 70 °C for subsequent analysis. Urinary CCL14 was detected using an enzyme-linked immunoassay kit (Conlon Biologicals, testing instrument: enzyme standard analyzer: Rayto RT-6100), the operator was blinded to the experimental study content and the kit instruction was strictly followed.

Renal ultrasound was performed within 12 h of admission using the CX50compactXtreme Ultrasound System (Philips, USA), and the renal measurements were performed via the posterior-lateral approach, preferring the right kidney, or if the right kidney was unavailable, the left kidney was measured, choosing either the interlobular or the arcuate artery. RRI was calculated as follows: RRI = (peak systolic rate−end-diastolic rate)/peak systolic rate, measured thrice and then averaged.

### Statistical analyses

Normally distributed measures were expressed using the mean ± standard deviation (X ± s), and the non-normally distributed measurements were expressed as the median (interquartile range) [Median (IQR)]. A T-test or rank-sum test was performed for comparison between the groups. Count data were expressed as frequencies (N) and percentages (%), and the Chi-square test or Fisher's exact probability method was applied for comparison between the groups. The receiver operating characteristic curve (ROC) was plotted to assess the diagnostic performance of RRI and CCL14 for predicting persistent AKI; the optimal threshold was defined with the Youden index. *P* < 0.05 was considered to indicate statistical significance. Statistical analysis was performed using the R software (version 4.2.1).

## Results

### Study population

**Fig. 1:**
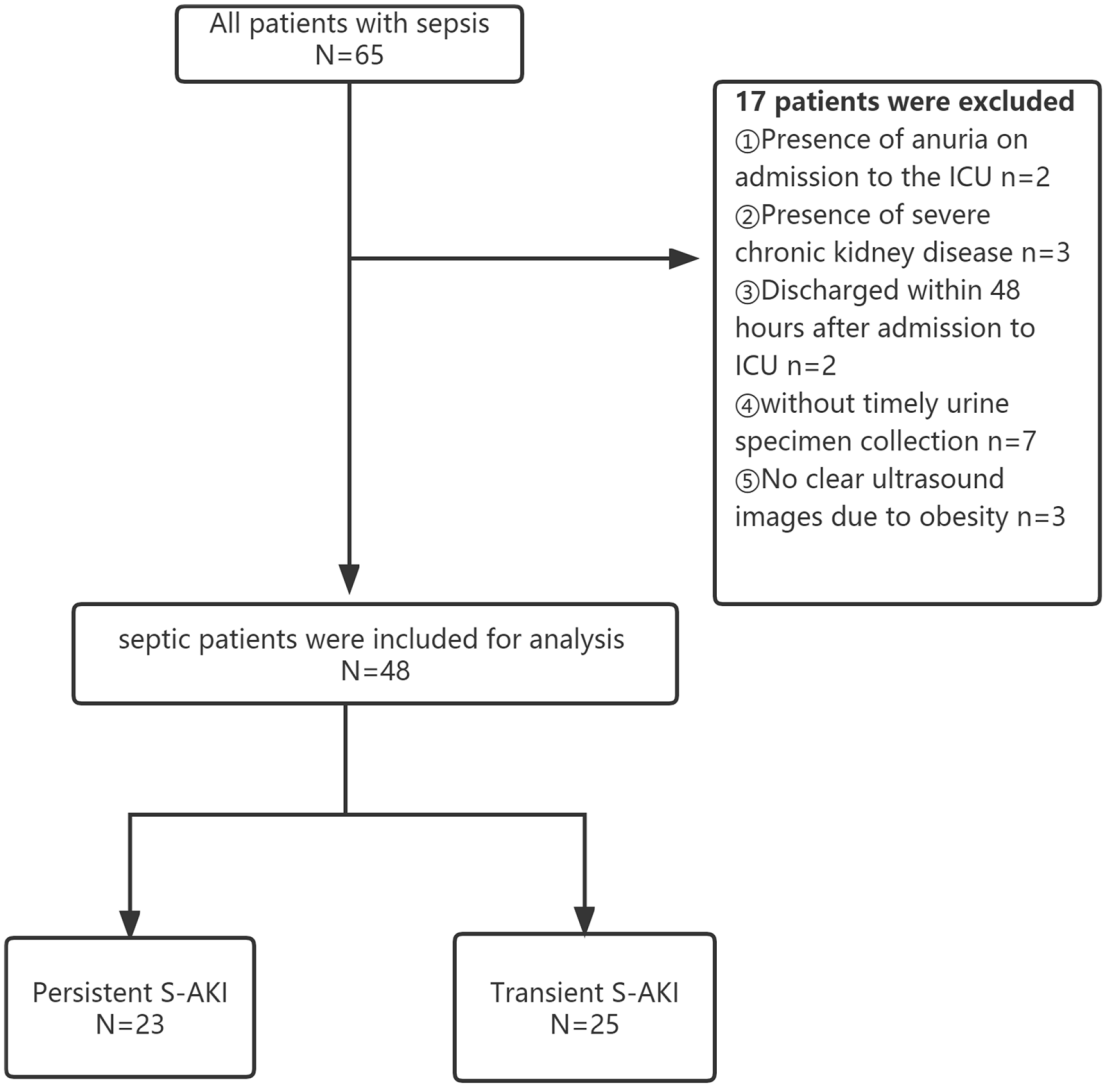
Flowchart of patients. *S-Akl* Sepsis-Associated Acute Kidney Injury

We screened 65 patients during the study period and excluded 17 patients, which included 2 patients with anuria at the time of ICU admission, 3 patients with severe chronic kidney disease (CKD stage 5), 2 patients who were discharged within 48 h of ICU admission, 7 patients who had CRRT immediately after ICU admission without timely retention of urine specimens, and 3 patients for whom clear ultrasound images could not be obtained due to obesity. Finally, based on the pre-defined disease condition, 48 patients were screened. Of which, 23 (48%) had persistent S-AKI and 25 (52%) had transient S-AKI (Fig. 1). The characteristics of the included patients are described in Table 1. A total of 18 (37.5%) patients presented with stage I AKI, 18 (37.5%) with stage II AKI, and 12 (25%) with stage III AKI. Over the period of study, 25 patients were ventilated, 28 patients were treated with vasoactive drugs, and 8 patients were treated with continuous renal replacement therapy. The median length of ICU stay was 9 days (7–13 days), and the ICU mortality rate was 12.5%.

**Table 1:** Characteristics of the studied population and differences according to sepsis-associated acute kidney injury reversibility

	Transient S-AKI	Persistent S-AKI	*p*. overall
	*N*=25	*N*=23	
Patient characteristics			
Sex, male, *n* (%)	13 (52.0%)	12 (52.2%)	1.000
Age, yr, mean ± sd	64.6 ± 12.9	66.4 ± 17.1	0.694
Chronic comorbidities			
Hypertension, *n* (%)	12 (48.0%)	14 (60.9%)	0.546
Diabetes, *n* (%)	5 (20.0%)	8 (34.8%)	0.409
Chronic Kidney Diseases, *n* (%)	1 (4.00%)	4 (17.4%)	0.180
Cerebrovascular disease, *n* (%)	2 (8.00%)	4 (17.4%)	0.407
Cardiovascular disease, *n* (%)	6 (24.0%)	6 (26.1%)	1.000
Cancer, *n* (%)	2 (8.00%)	5 (21.7%)	0.237
Characteristics at inclusion			
Mean arterial pressure, mm Hg, mean ± sd	78.8 (20.3)	85.0 (16.0)	0.249
Lactate (mmol/L), median (IQR)	1.60 [1.20; 1.90]	3.10 [2.70; 4.05]	< 0.001
Simplified Acute Physiology Score II, mean ± sd	55.6 ± 15.6	65.3 ± 10.9	0.016
Sequential Organ Failure Assessment, median (IQR)	8.00 [6.00; 9.00]	10.0 [8.00; 14.0]	0.009
Renal function at inclusion			
Diuresis (mL/kg/hr), median (IQR)	1.00 [0.39; 2.14]	0.78 [0.47; 1.54]	0.984
Serum creatinine (µmol/L), median (IQR)	116 [96.8; 159]	161 [132; 284]	0.025
Serum creatinine_48hours (µmol/L), median (IQR)	80.3 [63.5; 90.7]	167 [129; 240]	< 0.001
AKI Stage			0.004
KDIGO 1, *n* (%)	15 (60.0%)	3 (13.0%)	
KDIGO 2, *n* (%)	6 (24.0%)	12 (52.2%)	
KDIGO 3, *n* (%)	4 (16.0%)	8 (34.8%)	
Organ support and treatment at inclusion			
Mechanical ventilation, *n* (%)	12 (48.0%)	13 (56.5%)	0.763
Vasoactive drugs, *n* (%)	17 (68.0%)	11 (47.8%)	0.261
Continuous renal replacement therapy, *n* (%)	1 (4.00%)	7 (30.4%)	0.020
CCL14 and renal resistive index			
Renal resistive index H12, mean ± sd	0.67±0.06	0.72 ±0.07	0.020
CCL14 T0, median (IQR)	597 [524; 652]	609 [567; 685]	0.403
CCL14 T6, median (IQR)	523 [470; 572]	547 [483; 605]	0.179
CCL14 T12, median (IQR)	480 [409; 506]	483 [403; 525]	0.828
CCL14 T24, median (IQR)	420 [307; 504]	376 [301; 450]	0.286
Prognosis			
Length of ICU stay (d), median (IQR)	9.00 [7.00; 11.0]	14.0 [8.00; 15.5]	0.020
Death in ICU, *n* (%)	2 (8.0%)	4 (17.4%)	0.407

*AKI* acute kidney injury, *IQR* interquartile range, *KDIGO* Kidney Disease Improving Global Outcomes, *CCL14* C–C motif chemokine ligand 14

### Comparison of patients with transient and persistent S-AKI

**Fig. 2:**
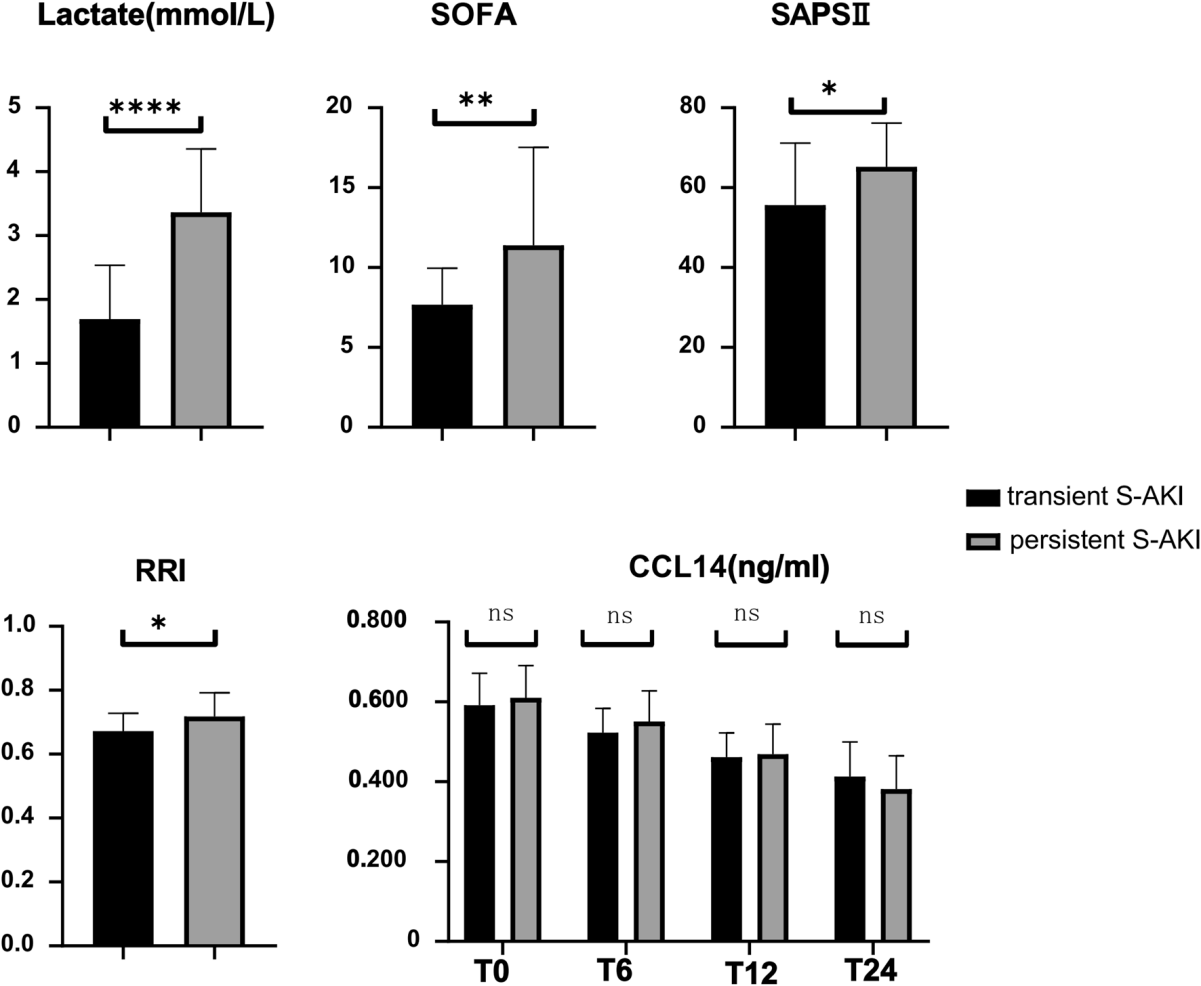
Comparison of transient and persistent S-AKI patients in lactate, SOFA, SAPS II RRI and CCL14, *S-AKl* Sepsis-Associated Acute Kidney lnjury, *SOFA* Sequential Organ Failure Assessment, *SAPS II* Simplified Acute Physiology Score 11, *CCL14* C–C motif chemokine ligand 14, *RRl* Renal resistance index, *p < .OS; ns: No statistical significance

It was observed that, at inclusion, the lactate levels and the SAPS II and SOFA scores were higher in patients with persistent S-AKI (Fig. 2) than in those with transient S-AKI. In patients with persistent S-AKI, CRRT was conducted at a higher rate (30.4 vs 4.0% *p* = 0.020) relative to that in the patients with transient S-AKI. Moreover, the ICU stay was also significantly longer (14 days [8–15.5 days] vs 9 days [7–11 days] *p* = 0.020) for persistent patients than the transient ones. ICU mortality was not significantly different between the 2 groups (17.4 vs 8% *p* = 0.407) (Table 1).

### Performance of urinary CCL14 to predict persistent S-AKI

**Fig. 3:**
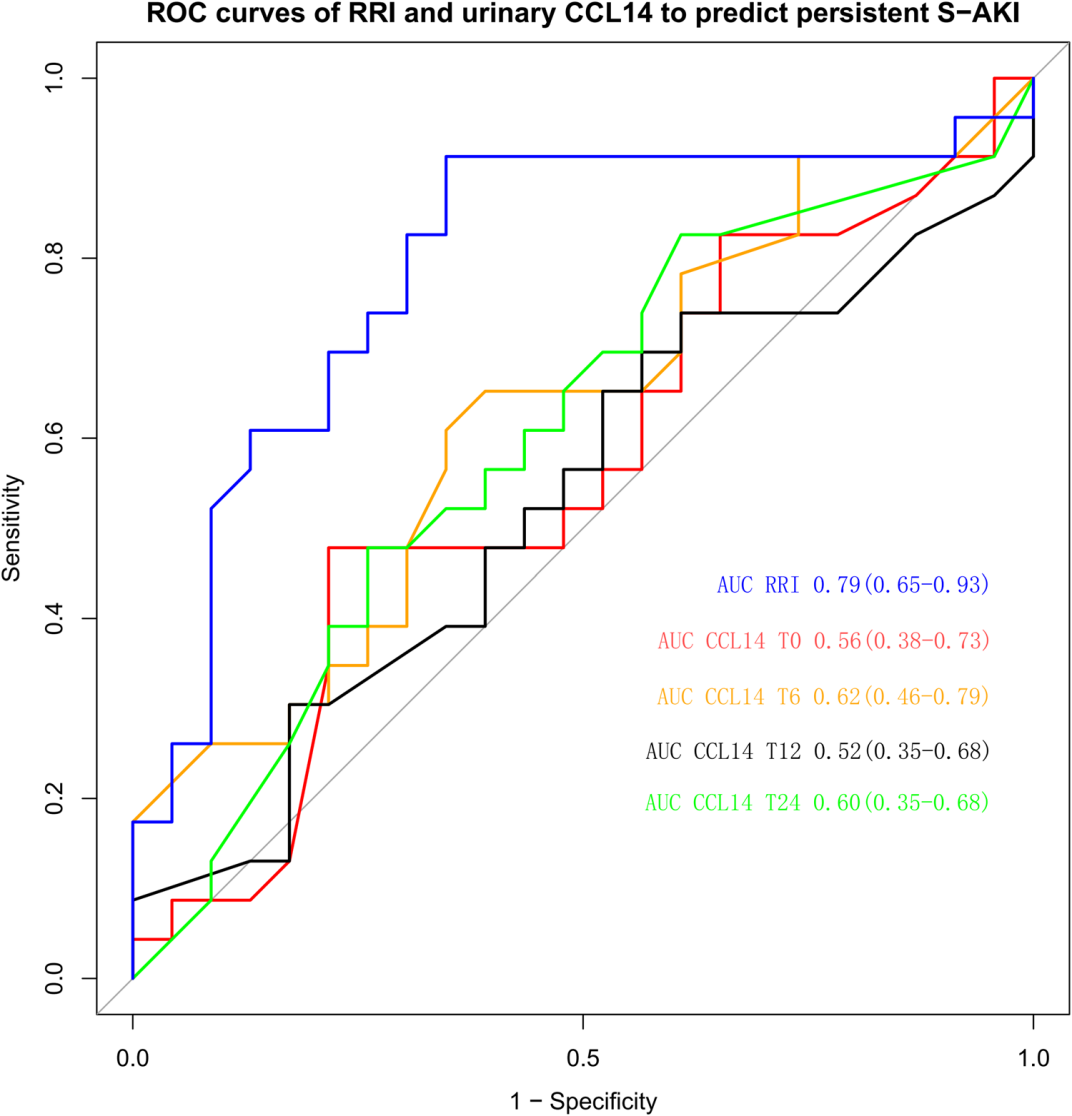
ROC curves of RRI and urinary CCL14 to predict persistent S-AKL

Urinary CCL14 did not differ significantly between the two groups at any time points, and the level of CCL14 gradually decreased within 24 h of admission to the ICU (Table 1, Fig. 2). The performance of urinary CCL14 in predicting persistent S-AKI was poor, with, respectively, an area under the ROC curve (AUC ROC) of 0.57 (95% CI 0.45–0.68), 0.58 (95% CI 0.47–0.69), 0.61 (95% CI 0.50–0.72), and 0.57 (95% CI 0.46–0.68) at T0, T6, T12, and T24 (Fig. 3, Table 2).The subgroup analysis of patients with S-AKI KDIGO stage II–III showed that the CCL14 level of persistent severe S-AKI was high at T0(*P* = 0.011), When CCL14 ≥ 0.603 ng/ml at T0, the AUC for predicting persistent severe S-AKI was 0.85 (95% CI 0.64–1.00), the sensitivity was 100%, and the specificity was 78%(Supplement 1).

**Table 2:** Performance of renal resistive index and CCL14 for predicting persistent acute kidney injury in the overall population

Performance	Renal Resistive Index ≥ 0.695	CCL14 T0 ≥ 0.658	CCL14 T6 ≥ 0.530	CCL14 T12 ≥ 0.451	CCL14 T24 ≥ 0.358
Sensitivity (%)	91	78	60	48	74
Specificity (%)	65	47	65	65	48
Positive predictive value (%)	91	60	63	58	59
Negative predictive value (%)	88	68	62	55	65
Positive likelihood ratio	2.60	1.47	1.71	1.37	1.42
Negative likelihood ratio	0.14	0.47	0.62	0.80	0.54
Youden`s index	1.56	1.25	1.25	1.13	1.22
Area under the receiver operating characteristic curve a	0.79 (0.65–0.93)	0.56 (0.38–0.73)	0.63 (0.46–0.79)	0.53 (0.35–0.68)	0.60 (0.43–0.77)

*CCL14* C–C motif chemokine ligand 14

^a^Values are given with 95% CI

### Performance of the RRI to predict persistent S-AKI

RRI was statistically higher in individuals with persistent S-AKI than in those with transient S-AKI (0.72 ± 0.07 vs 0.67 ± 0.06; *p* = 0.020) (Table 1, Fig. 2). The AUC ROC for RRI for persistent S-AKI patients was 0.79 (95% CI, 0.65–0.93) (Fig. 3). RRI ≥ 0.695 was used for the prediction of persistent S-AKI with a sensitivity of 91.30% (95% CI 70–98%) and a specificity of 65.20% (95% CI 43–83%) (Table 2). The AUC ROC of RRI was better than that of urinary CCL14 (*p* < 0.01).

## Discussion

Early detection of persistent S-AKI in the ICU setting is challenging but of critical clinical importance. When compared to transient S-AKI, persistent S-AKI is more severe and associated with poor clinical outcomes [25]. Early detection of persistent S-AKI can, therefore, help in optimizing treatment, such as the rapid restoration of renal perfusion, fluid restriction, avoidance of nephrotoxic drugs, or early CRRT [25].

In the present study, we evaluated the predictive ability of urinary biomarkers CCL14 and RI for persistent S-AKI. We found that the CCL14 levels measured at different time points within 24 h of ICU admission did not differ significantly between the persistent S-AKI and transient S-AKI groups, but RRI measured within 12 h of ICU admission performed well in predicting persistent S-AKI and was significantly better than urinary CCL14 for this purpose.

Different urobiological markers such as fractional excretion of urinary sodium (FENa) and fractional excretion of urinary urea (FEU) have been used for differentiating transient from persistent AKI. However, their reliability in the ICU setting remains unknown [26–28]. Therefore, there is a need to determine and validate new biomarkers to achieve early diagnosis of persistent AKI. Hoste et al*.*[21] described 331 patients with stages II and III AKI and identified and validated potential biomarkers to identify patients with persistent severe AKI (stage III) lasting for ≥ 72 h. Of all potential biomarkers associated with AKI persistence (i.e., apoptosis, necrosis, endothelial injury, cell–cell and cell–matrix adhesion, cytoprotection, oxidative processes, cell cycle regulation, inflammation, kidney injury, immune function, and fibrosis), the researchers found that CCL14 had the highest ability to predict AKI persistence with an AUC of 0.83, which outpaced other biomarkers markers (i.e., urinary Chitinase3-Like1 and plasma cystatin). Similarly, Bagshaw et al*.* [22] externally validated CCL14 to predict persistent AKI. They assessed 195 patients with KIDGO stages II–III. The AUC for urinary CCL14 to predict persistent KIDGO stage III AKI was found to be 0.81 (95%CI, 0.72–0.89), and it was found that the higher the urinary CCL14 value, the higher the risk of persistent KIDGO stage III AKI. Therefore, it must be acknowledged that both the discovery study by Hoste et al. and the external study by *Bagshaw *et al. had heterogeneity in the study population and included patients with KIDGO stages II–III. It is, therefore, unknown whether the same diagnostic efficacy of urinary CCL14 exists for different AKI subtypes and patients with different AKI stages.

Our cumulative results showed that CCL14 was not effective in predicting persistent S-AKI without differentiation of severity, as suggested by previous studies that CCL14 was mainly used to predict persistent severe AKI (stages II–III) [21, 22, 29]. We also performed a subgroup analysis to further investigate the diagnostic value of CCL14 in sepsis-associated persistent severe AKI, and the results suggested that CCL14 could predict persistent severe S-AKI at T0, but not after 6 h of ICU admission, which may be related to the decay of the marker, further studies are needed to explore the change pattern of CCL14 over time.

Moreover, the use of RRI in the detection of persistent AKI is well reported. Doppler ultrasound is increasingly being used in the ICU owing to its simplicity, rapidity, ease of application, and noninvasiveness. Doppler ultrasound-based RRI can be used to measure renal vascular resistance and renal blood flow [12, 30]. Some researchers have demonstrated that RRI can facilitate the early detection of AKI in ICU patients [12–14]. In preliminary studies conducted in the ICU, RRI was found to be higher in patients with persistent AKI at the time of admission. These researches suggest that RRI has a good ability to differentiate between transient and persistent AKI [14, 16]. Meta-analyses have reported similar results [17]. However, most of these past studies were single-center, small-sample studies with significant heterogeneity between the studies. In addition, a recent multi-center study of 233 patients with AKI reported conflicting results, with RRI performing poorly in predicting persistent AKI [31]. We evaluated the effect of RRI in predicting S-AKI. Our main objective was to assess the diagnostic value of RRI in S-AKI. We found that RRI measured within 12 h of admission to the ICU performed well in predicting persistent S-AKI, with an RRI ≥ 0.695, a sensitivity of 91.30% (95% CI 70–98%), and a specificity of 65.20% (95% CI 43–83%).The differences observed for the values of RRI between the two groups of patients, although significant, are too small to have clinical usability; the lack of an effective cut-off limits the use of this technique to identify patients at risk with certainty. However, the study suggests that higher resistance index values are indicative of a higher risk of persistent S-AKI; the usefulness of the RRI is to be understood in the follow-up of the patient, to help the clinician predict the course of the pathology over time. These results prove the efficacy of RRI in detecting persistent S-AKI, which warrants further exploration.

Our study has several advantages. First, we defined AKI and persistent renal dysfunction using the latest consensus guidelines [5]. Second, we measured both the RRI and the diagnostic value of the novel biomarker CCL14 for persistent S-AKI. Third, our study population was selected for the subtype of S-AKI. According to the latest published literature, this is the first evaluation of the diagnostic value of CCL14 for persistent S-AKI. Moreover, we evaluated the effect of different measurement times on its diagnostic efficacy. However, our study has limitations. First, this study was a single-center study with small sample size; the work is conditioned by the reduced number of enrolled patients and the lack of a power analysis. Second, the identification and measurement of RRI by Doppler ultrasound was largely operator dependent. To control inter-observer variability, two investigators performed renal Doppler examinations, but we did not assess the potential inter-operator RRI variability. However, past studies have shown that brief training in renal Doppler ultrasound for clinicians unfamiliar with this technique can produce both feasible and reliable results [32].

## Conclusions

Our study evaluated two different options to detect persistent S-AKI, namely technique based and using a biomarker. The RRI showed a good predictive performance in persistent S-AKI, especially in critically ill patients; however, urinary CCL14 could not discriminate between transient and persistent S-AKI. Although the results are promising, validation using a higher sample size will be more beneficial.

## Supplementary Information

Below is the link to the electronic supplementary material.

Supplementary file1 (DOCX 189 kb)

## Data Availability

Not applicable.
