# Supplementary material for: Predictability performance of urinary C–C motif chemokine ligand 14 and renal resistive index for persistent sepsis-associated acute kidney injury in ICU patients
Source: Int Urol Nephrol. 2023 Feb 17;55(8):1995–2003. doi: 10.1007/s11255-023-03511-6 (PMC10329624; doi:10.1007/s11255-023-03511-6)

|  | Non-persistent severe S-AKI | Persistent severe S-AKI | p.overall |
| --- | --- | --- | --- |
|  | N=9 | N=9 |  |
| CCL14 T0, median (IQR),pg/ml | 588 [521-597] | 685 [685-687] | 0.011 |
| CCL14 T6, median (IQR),pg/ml | 525 [470-567] | 559 [470-625] | 0.331 |
| CCL14 T12, median (IQR),pg/ml | 489 [411-506] | 506 [488-525] | 0.534 |
| CCL14 T24, median (IQR),pg/ml | 376 [354-477] | 467 [301-504] | 0.476 |

Supplement 1 urinary CCL14 to predict persistent severe S−AKI

A.Comparison of CCL14 level between non persistent severe S-AKI and persistent severe S-AKI

B.ROC curves of urinary CCL14 at T0 to predict persistent severe S−AKI


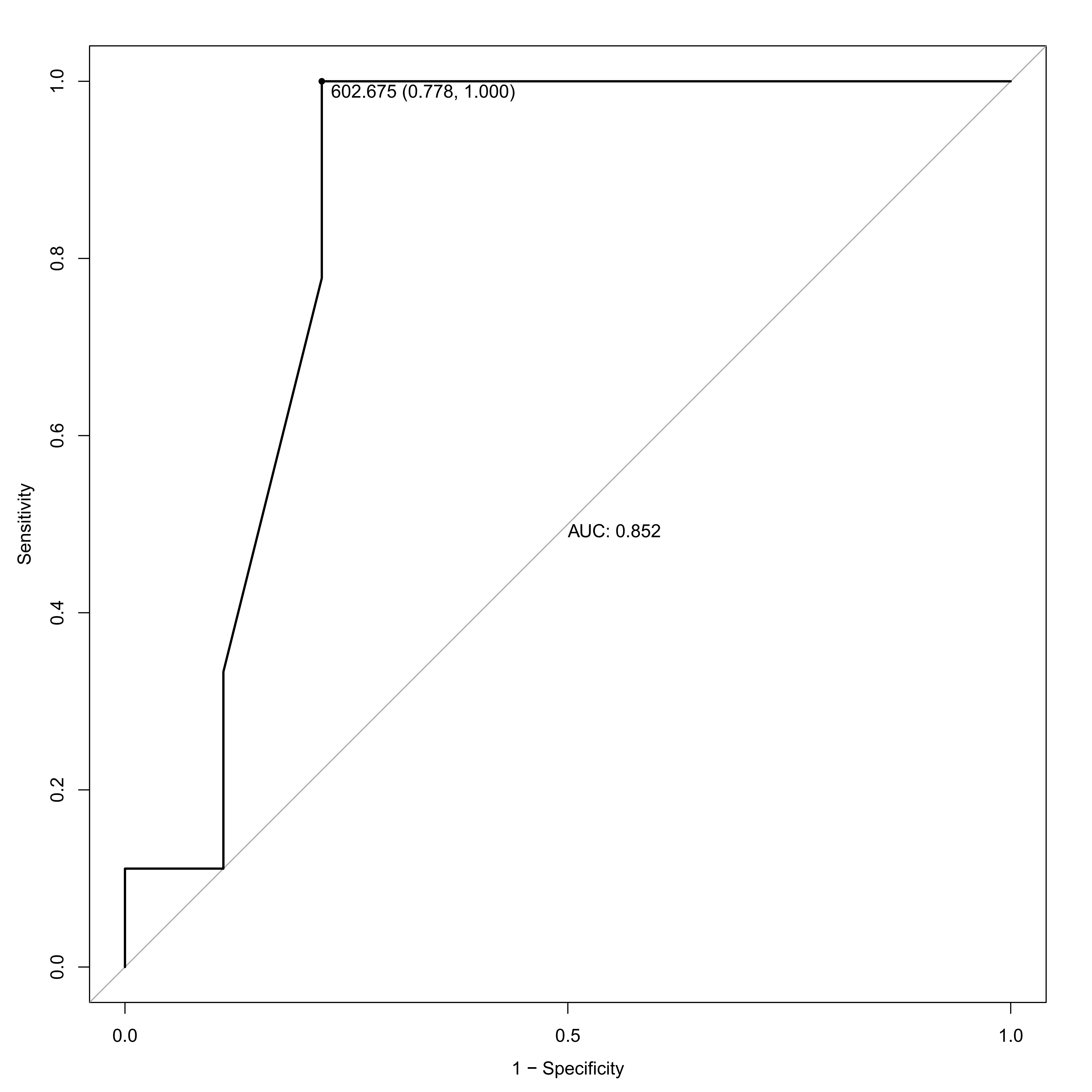

Supplement: Supplementary file 1 — Supplementary file1 (DOCX 189 kb) [file 11255_2023_3511_MOESM1_ESM.docx]
